# Distinct Mechanisms of Contextual Modulation for Dynamic Natural Scenes in Motion- and Scene-Selective Cortex

**DOI:** 10.1152/jn.00063.2026

**Published:** 2026-06-10

**Authors:** Merve Kiniklioglu, David Pitcher, Daniel Kaiser

**Affiliations:** 1 Neural Computation Group, Department of Mathematics and Computer Science, Physics, Geography, Justus Liebig University Gießen, 35392 Gießen, Germany; 2 Department of Psychology, University of York, Heslington, York, YO10 5DD, UK; 3 Cluster of Excellence ”The Adaptive Mind”, Universities of Giessen, Marburg, and Darmstadt, 35392 Gießen, Germany; 4 Center for Mind, Brain and Behavior (CMBB), Universities of Giessen, Marburg, and Darmstadt, 35032 Marburg, Germany; 5 Center for Applied Computer Science and Data Science (ZAD), Justus Liebig University Gießen, 35392 Gießen, Germany

**Keywords:** contextual modulation, fMRI, motion perception, multivariate pattern analysis, scene perception, surround suppression

## Abstract

Neural responses in visual cortex to a central stimulus are modulated by its surrounding context. Although this contextual modulation is well established for simple stimuli, how it manifests during dynamic naturalistic scene perception remains unclear. Using fMRI, we examined how motion congruence and categorical similarity between central and surrounding scenes shape contextual modulation across the visual hierarchy. Central and surrounding scenes systematically varied in categorical similarity (identical exemplar, different exemplar of the same basic-level category, different basic-level category, or different superordinate category) and in motion direction (same or opposite direction). Neural responses were measured in primary visual cortex (V1), motion-selective cortex (hMT+), and scene-selective occipital and parahippocampal place areas (OPA and PPA). hMT+ showed robust motion-dependent contextual modulation, with stronger suppression for same-direction motion. V1 responses were sensitive to low-level similarity between center and surround, with reduced responses when center and surround were physically identical and moved in the same direction. In contrast, scene-selective regions (OPA and PPA) did not show univariate modulation by motion congruence or categorical similarity, but multivoxel activity patterns reflected the categorical relationship between center and surround. Multivariate analyses further showed that decoding accuracy in OPA and PPA increased as categorical similarity decreased. Together, these results demonstrate that contextual modulation in natural vision differs across visual regions, reflecting sensitivity to motion relationships in hMT+, low-level similarity in V1, and higher-level categorical structure in scene-selective cortex. These findings highlight how contextual influences operate at multiple levels of visual representation to support the processing of dynamic natural scenes.

**Figure:**
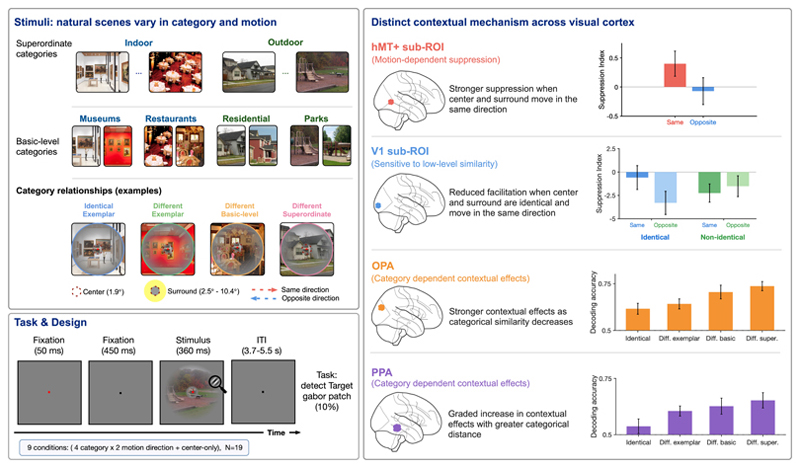

## Introduction

Sensitivity to visual stimuli is strongly influenced by the surrounding context. A classic example of such contextual influences is surround modulation, in which the response of a neuron to a stimulus presented in the receptive field (RF) center is affected by stimuli presented in the surround. This modulation can manifest as suppression (*surround suppression*) or facilitation (*surround facilitation*) [1–11]. These effects have traditionally been attributed to antagonistic center–surround interactions within the visual cortex, particularly in motion-selective regions such as the middle temporal area (MT) [e.g., MT hypothesis; see 12–18].

Center-surround interactions have been observed across multiple stages of the visual hierarchy. Surround modulation has been demonstrated in retinotopic areas of early visual cortex (V1-V4) as well as in motion-selective regions such as MT and MST, suggesting that it reflects a canonical computation within the visual system [19–23]. Importantly, the strength and form of these interactions depend on stimulus properties. Both neural [6, 15, 16, 18, 24] and behavioral studies [13–16, 25] show that low-level features such as size, contrast, and motion direction systematically influence the surround modulation. Typically, feature similarity between center and surround increases suppression, whereas dissimilarity reduces it and can even reverse the effect into facilitation. For example, neurons responding to a central grating are strongly suppressed by iso-oriented surrounds but can be facilitated when the surround is orthogonal [5, 26, 27]. A comparable pattern is observed for motion, where suppression is typically stronger when the center and surround drift in the same direction than when they move in opposite directions [5, 18, 23, 28–30].

Contextual influences extend beyond classical surround modulation paradigms. Functional MRI studies show that responses in early visual cortex are modulated by context during contour integration, with activity increasing when aligned contours must be detected within cluttered backgrounds [31, 32]. Beyond early visual cortex, similar effects have also been observed in higher-level object-selective regions. For example, activity in the lateral occipital complex (LOC) increases when spatially distributed visual elements are grouped into coherent contours or shapes [32, 33]. Together, these findings indicate that contextual modulation operates across multiple stages of visual processing and supports the integration of increasingly complex visual structure.

Importantly, the nature of contextual modulation varies across different regions of the visual system. In early visual areas, such effects are often linked to interactions within and beyond the classical receptive field [19, 20]. In contrast, contextual influences in higher-level visual regions are frequently associated with relationships between objects and scene elements within the broader visual environment [34–36]. These differences likely reflect the larger receptive fields in higher-level areas, which integrate information across broader regions of the visual field [37–39]. However, it remains an open question how these contextual interactions generalize to complex, dynamic scenes.

Our recent behavioral work using dynamic natural scenes provides evidence that such contextual interactions can depend on categorical relationships between center and surround scenes. Specifically, we found that surround suppression increased as the categorical similarity between the center and surround scenes increased [40]. These results are consistent with findings from scene categorization studies showing that categorically congruent contexts can facilitate recognition, whereas incongruent contexts impair performance [41, 42]. Consistent with prior work using simple stimuli [25, 30, 43], we also observed reduced suppression when the center and surround scenes moved in opposite directions. Together, these findings suggest that contextual modulation in natural vision may depend on both low-level visual features (e.g., motion direction) and higher-level relationships between scenes (e.g., categorical similarity).

While our behavioral findings demonstrate suppressive contextual interactions in dynamic natural scenes, neurophysiological studies using natural stimuli have reported both suppressive and facilitatory interactions under different experimental conditions. For example, in the macaque primary visual cortex (V1), suppression was stronger when the center and surround contained homogeneous natural images [44], whereas in the cat visual cortex, surrounds that are coherent with the central scene elicited facilitation [45]. Consistent with this, a human fMRI study shows that V1 responses to natural image fragments are enhanced when the surrounding context is coherent with the dominant scene [46]. Together, these findings indicate that contextual modulation is sensitive to the statistical properties, spatial organization, and coherence of natural stimuli. However, while prior work has demonstrated contextual modulation with static natural stimuli in V1, it remains unclear whether similar contextual influences are observed across different regions of the human visual system during the perception of dynamic natural scenes, and whether they are shaped by higher-level relationships such as categorical similarity between the center and surround scene.

To address this gap, we used functional magnetic resonance imaging (fMRI) to investigate how contextual relationships between central and surrounding scenes influence neural responses to dynamic natural scenes. We measured responses across multiple stages of visual processing, including primary visual cortex (V1), motion-selective cortex (hMT+), and the scene-selective occipital place area (OPA) and parahippocampal place area (PPA). This allowed us to directly compare how contextual modulation varies across regions associated with different aspects of visual scene processing, from low-level feature processing to higher-level scene representation.

Participants viewed central scenes presented together with surrounding scenes that varied in categorical similarity across four levels: identical exemplar, different exemplar from the same basic-level category, different basic-level categories within the same superordinate category, and different superordinate categories. We also manipulated motion congruence, comparing conditions in which the center and surround moved in the same versus opposite directions, to determine whether motion-dependent contextual effects observed with simple stimuli generalize to naturalistic scenes. By independently manipulating categorical similarity and motion congruence, our design systematically tests how contextual relationships at multiple levels of visual representation shape neural responses to dynamic natural scenes.

## Methods

### Participants

Twenty healthy volunteers (11 female, mean age = 26.7 years) participated in the study. One participant was excluded due to excessive head motion during scanning (see Preprocessing for motion-quality criteria), leaving a final sample of nineteen participants. The sample size (N = 19) is comparable to that used in previous studies investigating low-level surround modulation [14, 18, 27]. All reported normal or corrected-to-normal vision. Written informed consent was obtained before participation, and participants received monetary compensation. The study was approved by the Ethics Committee of Justus Liebig University Giessen. All experimental protocols were in accordance with the Declaration of Helsinki.

### Stimuli

Stimuli were panoramic videos created by moving static scene images behind a circular occluder (908 × 699 pixels) at a speed of 6°/s. The central image was presented through a 1.9°-diameter aperture, surrounded by an annulus ranging from 2.5° to 10.4°. To separate the center and surround, the area between them (1.9°-2.5°) remained un-stimulated. A cosine envelope was applied at the aperture boundaries to minimize sharp transitions at the edges.

**Figure 1:**
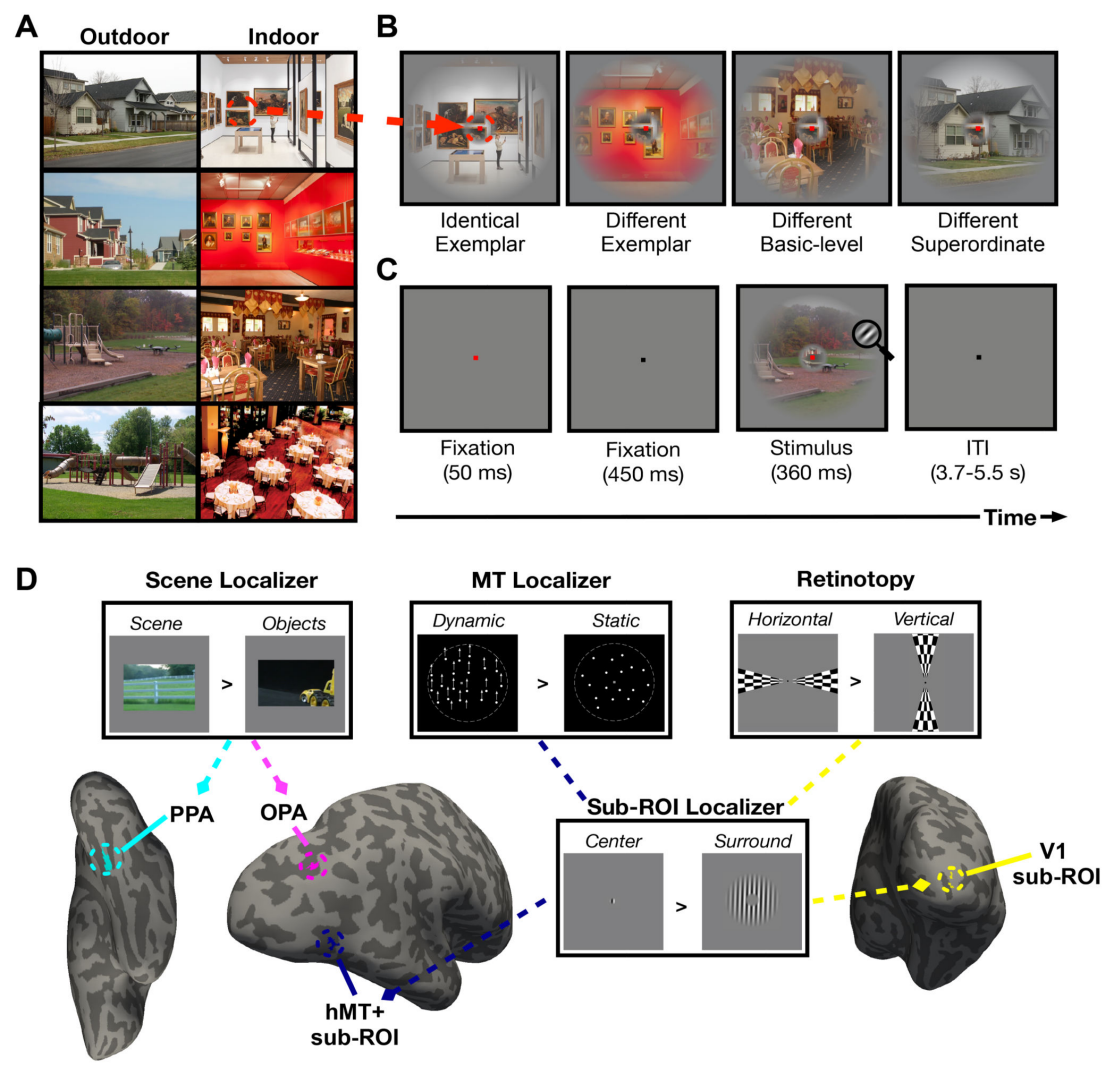
Stimuli, paradigm, and region of interest (ROI) definition. **A)** The natural scene images used to create the stimuli were drawn from two superordinate categories (indoor and outdoor) and two basic-level categories within each (restaurants, museums, parks, and residential areas), with two exemplars per basic-level category. **B)** Example stimuli for each category condition, shown from left to right: identical exemplar, different exemplar, different basic-level category, different superordinate category. **C)** Experimental design. Each trial began with a fixation point (500 ms), which briefly turned red to signal the upcoming stimulus, followed by presentation of a scene video (360 ms). Intertrial intervals varied randomly between 3.7 and 5.6 s. On 10% of trials, a high-contrast Gabor patch appeared at a random location within either the center or surround region, and participants reported its detection with a keypress. **D)** Functional localization of ROIs. ROIs were defined individually using independent functional localizers. hMT+ was identified with the *dynamic > static* contrast (*p*_uncorrected_ < 0.001), and V1 with the *horizontal > vertical* contrast (*p*_uncorrected_ < 0.001). Sub-ROIs in hMT+ and V1 were further defined using a center–surround localizer (*center > surround* ; *p*_uncorrected_ < 0.0001). Scene-selective regions (OPA, PPA) were identified using a *scene > object* contrast (*p <* 0.05). Because no reliable center–surround effects were observed, full ROIs were used for these regions. The figure shows a representative cortical surface from a single subject’s right hemisphere, illustrating ROI locations.

Scene images were selected from two superordinate categories (indoor: restaurants, museums; outdoor: parks, residential areas), each featuring two basic-level categories, with two exemplars per basic-level category, resulting in a total of eight scene images. The images shown in Figure 1 illustrate the stimulus set. The center scenes were always presented with a surrounding scene, except in center-only trials where the central stimulus appeared alone (see below). The resulting center–surround stimuli varied in their categorical relationship between center and surround, with four conditions: identical exemplar condition, where the center and surround images were identical; different exemplar condition, where the center and surround images belonged to the same basic-level category (e.g., museums) but were not identical; different basic-level category condition, where the center and surround images belonged to the same superordinate category (e.g., indoor) but were from different basic-level categories; and different superordinate category condition, where the center and surround images belonged to different superordinate categories (see Figure 1). Motion congruency was manipulated with two conditions: in half of the trials, the center and surround scenes moved in the same direction, and in the other half, they moved in opposite directions.

### Experimental Paradigm

We used a mixed event-related design in which center-only and center+surround trials were presented within the same run (Figure 1). In total, nine trial types (four category conditions × two motion directions, plus the center-only condition) were presented in randomized order. Center-only trials served as a baseline for quantifying contextual modulation by the surround.

Each trial began with a fixation point (500 ms), followed by a scene video (360 ms) on a mid-gray background. Participants were instructed to maintain fixation at the center of the display. Intertrial intervals varied randomly between 3.7 and 5.6 seconds, sampled from a uniform distribution. To ensure that participants attended to both center and surround, on 10% of trials a small Gabor patch (spatial frequency = 1 cycles/°, diameter = 0.7°, contrast = 98%) appeared at a random location within either the center or surround region. Participants reported its detection with a keypress, with a mean accuracy of 92.2% (SD = 7.6%). Each condition was presented eight times per run, and participants completed five runs. Each run lasted approximately 10 minutes, and short breaks were provided between runs.

### Data Acquisition

MRI data were acquired on a 3T Siemens Magnetom Prisma scanner (Siemens Healthineers, Erlangen, Germany) equipped with a 64-channel head coil at the Bender Institute of Neuroimaging (BION), Justus Liebig University Giessen. High-resolution anatomical images were collected using a T1-weighted 3D sagittal MP-RAGE sequence (voxel size = 1 mm^3^ isotropic, 176 slices). Functional images were acquired with a T2*-weighted EPI sequence (TR = 1850 ms, TE = 30 ms, voxel size = 2.2 mm^3^, 58 slices). Visual stimuli were presented on a MR-compatible LED monitor (1920 × 1080 pixels, 120 Hz) positioned at the rear of the bore and viewed via a mirror mounted on the head coil at a distance of 140 cm. Stimuli were generated and presented using MATLAB (MathWorks, Natick, MA) and the Psychophysics Toolbox [47]. Participant responses were recorded using an MR-compatible fiber-optic response box. Each session began with an anatomical scan, followed by four localizer runs and five experimental runs, for a total duration of approximately 90 minutes.

### Localizer Runs

#### hMT+

The hMT+ area of each participant was defined using established methods [48]. Stimuli consisted of 200 randomly positioned white dots presented within a 12° aperture on a black background. In dynamic blocks, the dots moved along one of four trajectories: radial (expansion–contraction), angular (clockwise–counterclockwise rotation), horizontal (left–right), or vertical (up–down). Motion direction changed every 1.85 s to prevent adaptation. In static blocks, the dots were identical but remained fixed in position. Each block lasted 14.8 s, and dynamic and static blocks alternated eight times per run. Participants maintained central fixation and performed a color-change detection task in which they reported changes in the color of the fixation point.

#### V1

The V1 area of each participant was defined using a flickering checkerboard-patterned wedge paradigm, similar to established retinotopic mapping methods [49, 50]. Because we were specifically interested in the V1/V2 boundary, we used alternating horizontal and vertical wedges instead of rotating or expanding stimuli [51, 52]. The run consisted of 14.8-s blocks of horizontal and vertical wedges presented in alternation, repeated ten times in the run. As in the hMT+ localizer, participants maintained fixation and performed a color-change detection task.

#### OPA and PPA

Scene-selective areas were defined using 3-s movie clips from four categories: faces, scenes, objects, and scrambled objects [53, 54]. Each category included 60 clips. Face clips featured seven children filmed against a black background in close-up, showing only their faces as they danced or interacted with toys or with adults who remained out of frame. Scene clips were recorded in 15 different locations, primarily pastoral settings, and filmed from a slowly moving car. Object clips depicted 15 distinct inanimate items (e.g., mobiles, wind-up toys, toy planes, tractors, and rolling balls). Scrambled-object clips were generated by dividing each object movie into a 15 × 15 grid and spatially shuffling the resulting segments within each frame. Participants completed eight blocks per category, each consisting of six videos randomly sampled from the full set of 60 exemplars in that category. Although the same actor, scene, or object could appear more than once, the large stimulus pool made such repetitions unlikely. As in the previous localizer runs, participants maintained fixation and reported changes in the color of the fixation point.

### hMT+ and V1 Sub-ROI Localizer Run

Using an independent localizer run, we identified the voxels corresponding to the spatial location and size of the center stimuli as sub-ROIs within hMT+ and V1. This approach is commonly used to localize responses to low-level grating stimuli [14, 15, 18], but here we adapted it to ensure that the same spatially defined sub-ROIs could be applied in the analysis of naturalistic stimuli. In the localizer, participants viewed drifting high-contrast (98%) center and surround (i.e., annulus) gratings, matched in size and location to those used in the functional runs. The run consist of 14.8-s center blocks alternating with 14.8-s surround blocks, each separated by a 14.8-s blank period, repeated six times. As in the other localizers, participants maintained fixation and reported changes in the color of the fixation point.

### Data Analysis

#### Preprocessing

##### Localizer Runs

Localizer data were preprocessed and analyzed using the FM-RIB Software Library (FSL) (www.fmrib.ox.ac.uk/fsl) and Freesurfer [55–57]. High-resolution anatomical images were skull-stripped with BET. Preprocessing steps for functional images included motion correction with MCFLIRT, high-pass temporal filtering (100s), and BET brain extraction. Spatial smoothing (4 mm FWHM) was applied only for the scene localizer used to define scene-selective region of interests (ROIs); no smoothing was applied for V1, hMT+, or sub-ROI localizers. Each participant’s functional images were aligned to their own high-resolution anatomical image and registered to the standard Montreal Neurological Institute (MNI) 2-mm brain using FLIRT. The 3D cortical surface was constructed from anatomical images for each participant using FreeSurfer’s *recon-all* command for visualizing statistical maps, anatomical delineation, and identifying ROIs.

##### Experimental Runs

Experimental data were preprocessed and analyzed in SPM12 (www.fil.ion.ucl.ac.uk/spm/). Preprocessing steps included geometric distortion correction with the SPM FieldMap toolbox, motion correction and coregistration of functional volumes to each participant’s T1-weighted structural image. Structural images were segmented and normalized to MNI 2-mm standard space, and the same transformation was applied to the functional images. For ROI-based univariate analyses, the unsmoothed functional data were used to preserve spatial specificity of voxel responses. For multivariate pattern analyses (MVPA), functional images were spatially smoothed with a 6-mm FWHM Gaussian kernel to improve signal-to-noise ratio while minimizing spatial blurring [58, 59].

To assess data quality, head motion was quantified for each volume using framewise displacement (FD). Volumes with FD greater than 0.5 mm were flagged as motion outliers [60]. Runs with more than 30% flagged volumes were discarded [61]. Participants with two or more runs discarded were excluded [62]. One participant met this exclusion criterion and was removed from all analyses. Importantly, none of the remaining participants had any runs excluded; all results reported below are based on the remaining *N* = 19 participants.

### ROI Construction

For all ROI constructions, a general linear model (GLM) was applied using FSL’s FMRI Expert Analysis Tool (FEAT). The predicted fMRI response in each trial was computed assuming a double-gamma hemodynamic response function (HRF). Nuisance regressors for linear motion (derived from MCFLIRT) were also included in the model. For removing temporal autocorrelations, FILM prewhitening was applied [57]. Importantly, ROI definitions were based entirely on independent localizer runs and were therefore statistically independent from the experimental data used in subsequent analyses.

#### hMT+

The hMT+ ROI was defined individually for each participant using the motion localizer data. Statistical maps of the *dynamic > static* contrast were computed using FSL and thresholded at *p <* 0.001 (uncorrected) for each participant. The resulting activation maps were registered to each participant’s anatomical space and visualized on the cortical surface using FreeSurfer. Guided by the MT anatomical label from FreeSurfer, voxels showing stronger responses to dynamic than static dots near the ascending limb of the inferior temporal sulcus, consistent with the anatomical location of hMT+, were selected to define the hMT+ ROI and used as a mask for subsequent sub-ROI localization.

#### V1

The V1 ROI was defined individually for each participant using the retinotopic localizer data. Statistical maps for the *horizontal > vertical* meridian contrast were computed using FSL and thresholded at *p <* 0.001 (uncorrected) for each participant. Activation maps were registered to each participant’s anatomical space and visualized on the cortical surface using FreeSurfer. Guided by the V1 anatomical label from FreeSurfer, voxels located along the calcarine sulcus and consistent with the expected anatomical location of V1 were selected to define the ROI, which was used for subsequent analyses.

#### hMT+ and V1 sub-ROIs

To identify voxels corresponding specifically to the spatial location of the center stimulus, we analyzed data from the independent center–surround localizer run. Within the previously defined V1 and hMT+ ROIs, voxels showing significantly stronger responses to the center than to the surround grating (*center > surround*) were identified using a voxelwise threshold of *p <* 0.0001 (uncorrected). The resulting voxels were defined as V1 and hMT+ sub-ROIs and were subsequently used as masks for the analysis of the experimental runs.

#### OPA and PPA

For the scene-selective ROIs, we identified the voxels with the greatest scene versus face and object contrast using FSL. Clusters were identified using a voxelwise threshold of Z > 2.3 and a cluster-corrected significance threshold of *p <* 0.05, based on Gaussian random field theory [57]. The occipital place area (OPA) was localized as the scene-selective cluster on the lateral surface near the transverse occipital sulcus, and the parahippocampal place area (PPA) as the scene-selective cluster on the ventral visual cortex. The identified regions were used as OPA and PPA masks. Because the center vs. surround contrast did not yield reliable voxel-level responses in OPA and PPA, we used the full ROIs rather than sub-ROIs for the analysis of the experimental runs.

### Analysis of Experimental Runs

#### Univariate Analysis

The experimental runs were analyzed in SPM12 using a general linear model (GLM). For each run, separate regressors were specified for each of the nine conditions, and six motion parameters from realignment were included as nuisance regressors. Predicted BOLD responses were modeled by convolving event onsets with a double-gamma hemodynamic response function (HRF), and statistical parametric maps (SPMs) were generated to assess the main effect of each condition across runs. To quantify fMRI responses, percent signal change (PSC) values were calculated for each condition and used as the primary measure of fMRI response in all regions of interest.

Percent signal change (PSC) was estimated from condition-specific beta values extracted within each ROI. For each regressor, beta estimates were scaled by the corresponding regressor amplitude and normalized by the session-specific ROI baseline, with resulting PSC estimates averaged across runs within condition.

To determine whether the center-only (i.e., baseline) stimulus evoked reliable activity, PSC values in the center-only condition were first compared against zero using one-sample *t* -tests. In ROIs that either showed a reliable response to the center-only condition or were defined using center-selective sub-ROI selection, contextual modulation was quantified using the suppression index (SI) to characterize the direction of modulation, defined as

(1) $$SI=B_{Center}-B_{Center+Surround}$$

where *B*_._ denotes percent signal change (PSC) for the given condition. Negative SI values indicate facilitation, positive values indicate suppression, and an SI of 0 indicates no modulation. The use of SI allows us to determine the direction of contextual modulation, distinguishing between suppressive and facilitatory effects of the surround relative to the center-only response.

Contextual modulation was assessed by testing SI values against zero using one-sample, two-tailed Student’s *t* -tests with FDR correction for multiple comparisons. We then performed a two-way Analysis of Variance (ANOVA) on SI values with categorical similarity (four levels: identical exemplar, different exemplar, different basic-level category, different superordinate category) and motion-direction congruence (two levels: same-direction, opposite-direction) as factors for each sub-ROI using SPSS Version 25 (IBM Corp., Armonk, NY). Finally, post hoc paired-sample *t* -tests were used to examine differences between conditions following significant ANOVA effects.

In ROIs that either did not show a reliable response to the center-only condition or were defined without center-selective sub-ROI selection, analyses focused on direct comparisons among the center+surround conditions rather than subtraction-based metrics relative to the center-only baseline. In these regions, contextual effects were assessed using a two-way ANOVA on PSC values rather than SI values.

#### Multivariate Analysis

To investigate distributed patterns of activity, we performed ROI-based decoding using CoSMoMVPA [63] on condition-wise GLM beta estimates. For each participant and ROI (hMT+, V1, OPA, and PPA), beta images from each run and condition were assembled into voxel pattern vectors. Classification was implemented using linear discriminant analysis (LDA). Datasets were z-scored within training folds and class-balanced within chunks (runs). Cross-validation used a leave-one-run-out partitioning scheme, and mean accuracy across folds was taken as the decoding score.

To assess whether the presence of a surround stimulus alters distributed activity patterns, we first performed two-way decoding between the *center-only* and *center+surround* conditions. Two-way decoding quantifies the separability of multivoxel response patterns between center-only and center+surround conditions. Higher decoding accuracy reflects greater differences in these patterns, due to changes in overall response magnitude and/or voxelwise pattern configuration. Decoding accuracy was therefore used as an index of the strength of contextual effects, although it does not provide information about their direction (i.e., suppression vs facilitation). For each ROI, decoding was performed separately for all combinations of scene categories and motion directions. To assess categorical effects, data were merged across motion directions and decoding accuracies were compared across the four category conditions using a oneway repeated-measures ANOVA. To assess motion effects, data were merged across categories and decoding accuracy was compared between same- and opposite-direction conditions using paired-samples *t* -tests.

Because OPA and PPA were defined as full functional ROIs rather than center-selective sub-ROIs, decoding center-only versus center+surround trials in these regions could be confounded by differences in the spatial extent of stimulation. To address this, we performed an additional analysis restricted to the center+surround conditions, thereby holding stimulation extent constant across conditions. Specifically, we conducted four-way classification to distinguish among the four category conditions (identical exemplar, different exemplar, different basic-level category, and different superordinate category). Data were collapsed across motion directions to increase statistical power. This analysis tests whether multivoxel activity patterns encode information about the categorical relationship between center and surround scenes independently of differences in peripheral stimulation.

Statistical significance relative to chance was assessed using permutation testing. For each participant, condition labels were randomly shuffled within runs, and the decoding analysis was repeated 1,000 times to generate a null distribution of accuracies. For group-level inference, shuffled accuracies were averaged across participants for each permutation to obtain a null distribution of group mean accuracies. The observed group mean accuracy was then compared with this distribution, and the empirical p-value was computed as the proportion of permutations in which the shuffled mean exceeded the observed value (p < 0.05).

#### Visual Feature Analysis

To assess whether the observed effects could be explained by low-level image properties, we conducted a visual feature analysis quantifying low-level visual similarity between center and surround stimuli. Following prior work using hierarchical models of early visual processing [64], we used an HMAX-based representation to characterize low-level visual structure. For each image, responses were summarized into a fixed-length feature vector, and pairwise cosine similarity between L2-normalized C1 feature vectors was computed for all center–surround image pairs. Similarity values were then averaged within each categorical condition (identical exemplar, different exemplar, different basic-level category, different superordinate category).

## Results

### Univariate analyses reveal distinct patterns of contextual modulation across visual regions

**Figure 2:**
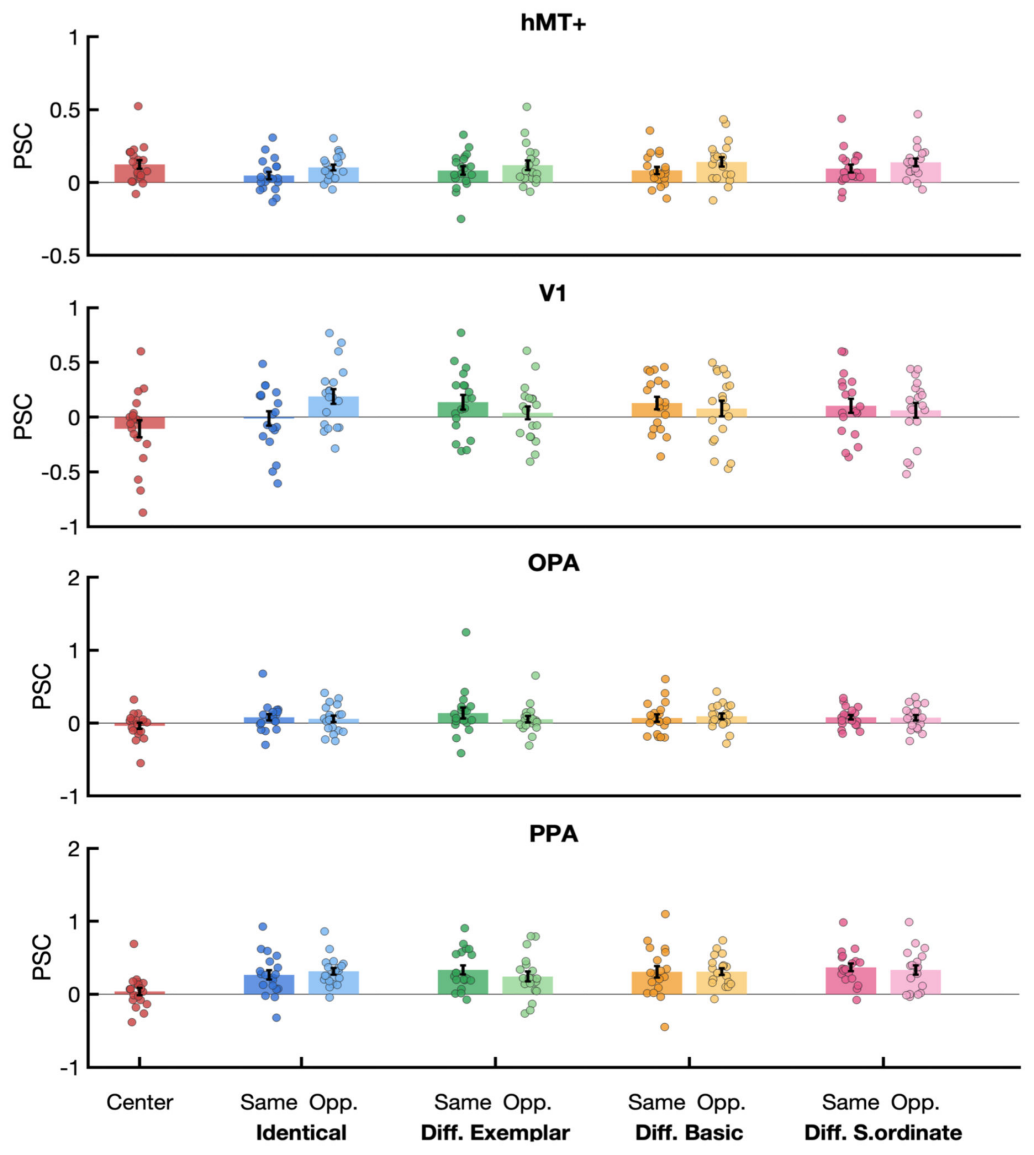
Univariate fMRI responses. Percent signal change (PSC) responses are shown across four category conditions (identical exemplar, different exemplar, different basic-level category, and different superordinate category), two motion-direction conditions (same and opposite), and the center-only condition. Results are displayed for hMT+, V1, OPA, and PPA. Circles represent individual participant values. Error bars represent the standard error of the mean (SEM).

We first tested whether the center-only condition evoked reliable responses in each ROI (see PSC values in Figure 2). The hMT+ sub-ROI showed a reliable center-only response (*t*(18)=4.11, *p <* 0.001). Contextual modulation was quantified using the suppression index (SI; see Methods).

**Figure 3:**
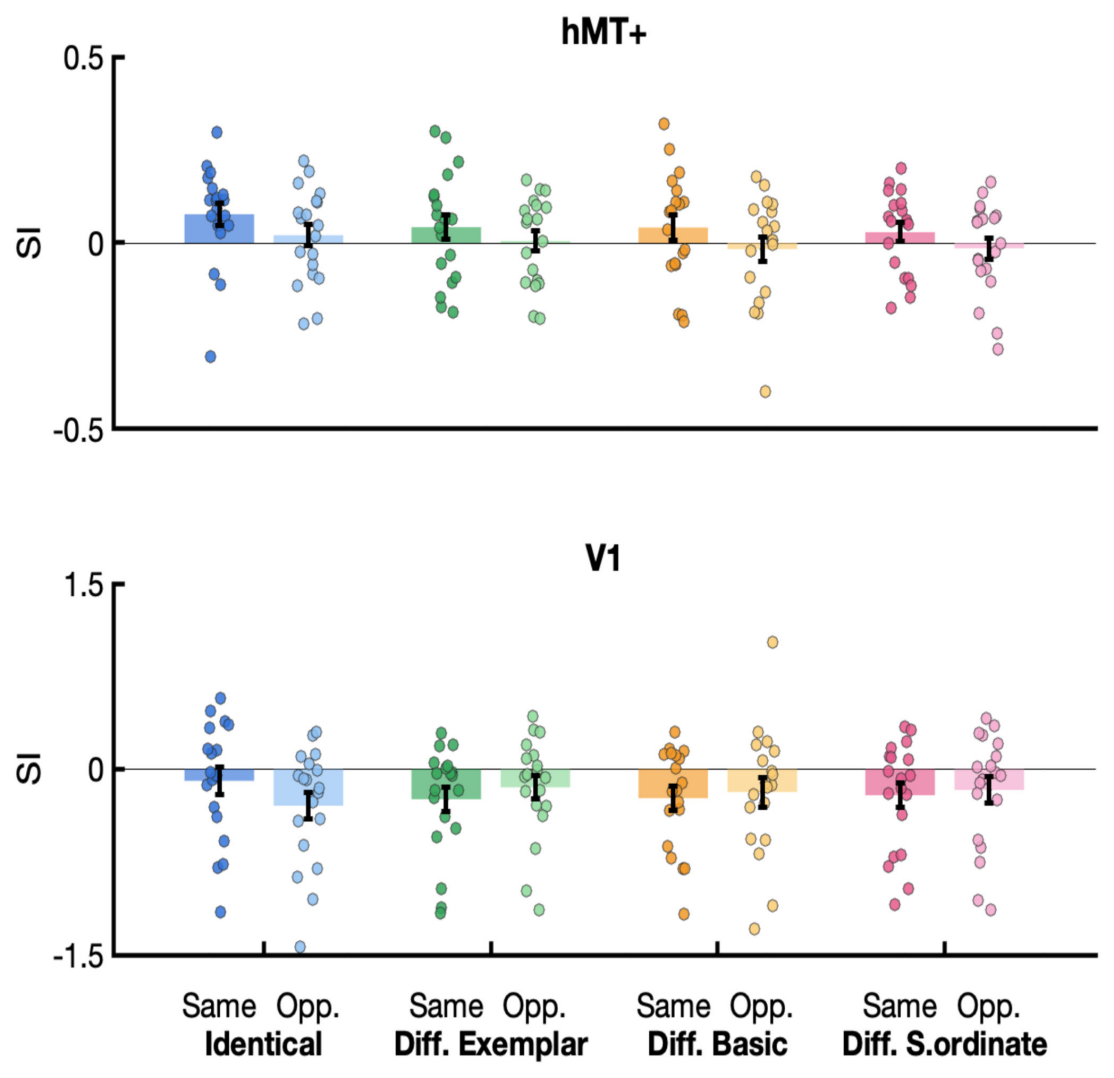
Univariate surround modulation effects. Suppression index (SI) values are shown across four category conditions: identical exemplar, different exemplar, different basic-level category, and different superordinate category, and two motion-direction conditions (same and opposite). Results are displayed for hMT+ and V1. Positive SI values indicate surround suppression; negative values reflect facilitation. Circles represent individual participant values. Error bars represent the standard error of the mean (SEM).

Figure 3 shows SI values for the hMT+ sub-ROI across the four categorical similarity conditions and two motion-direction conditions. SI values were significantly greater than zero only in the identical-exemplar condition for same-direction trials (*t*(18)=2.55, *p*_FDR_ = 0.04), indicating the presence of suppression by the contextual surround.

A two-way ANOVA revealed a significant main effect of motion direction (*F*(1,18)= 16.12, *p* = 0.001, *η*^2^ = 0.47), but no significant main effect of category (*F*(3,54)= 1.70, *p* = 0.18, *η*^2^ = 0.086), or interaction between category and direction (*F*(3,54)= 0.13, *p* = 0.94, *η*^2^ = 0.007). These results suggest that contextual suppression in hMT+ depends primarily on the relative motion direction between center and surround.

**Figure 4:**
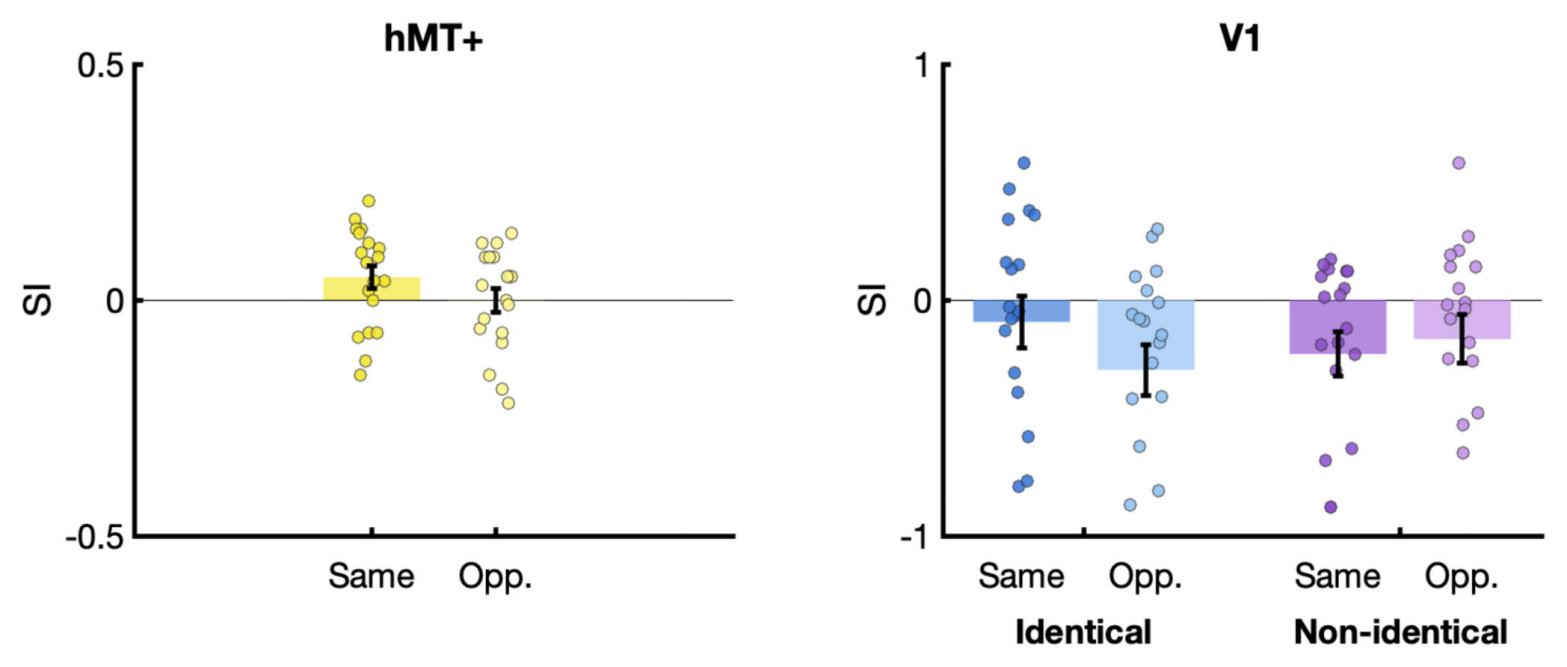
Averaged univariate contextual modulation effects (N=19) in hMT+ and V1. hMT+ SI values averaged across the four category conditions for same- and opposite-direction trials. V1 SI values for the identical exemplar condition versus the mean of the three other (non-identical) conditions (i.e., different exemplar, different basic-level category, and different superordinate category conditions), shown separately for same- and opposite-direction trials. Circles represent individual participant values. Error bars represent the standard error of the mean (SEM).

As expected, the categorical similarity of the center and surround had no effect on the suppression strength, consistent with hMT+ being primarily motion selective rather than sensitive to scene content. Because the categorical similarity neither modulated SI nor interacted with motion direction, SI values were averaged across the four category conditions (Figure 4). A paired-sample *t* -test on the averaged data confirmed that suppression was significantly stronger when the center and surround moved in the same direction than when they moved in opposite directions (*t*(18) = 4.02, *p* = 0.001). Together, these results demonstrate that hMT+ exhibits motion-dependent suppression under dynamic, natural scene conditions.

In V1 sub-ROI, responses to the center-only condition were not significantly different from zero (*t*(18)=-1.37, *p*=0.19). Contextual modulation was nevertheless quantified using the suppression index (SI) within these independently defined center-selective sub-ROIs. Figure 3 shows the SI values for the V1 sub-ROI across four scene-category conditions and two motion-direction conditions. SI values were significantly lower than zero in all conditions except the identical-exemplar condition for same-direction trials (*t*(18)= −0.09, *p*_FDR_ = 0.20).

Importantly, although center-only responses were not reliable in V1, responses varied systematically as a function of the relationship between center and surround stimuli. A two-way ANOVA revealed a significant interaction between motion direction and categorical similarity (*F*(3,54)=8.91, *p<* 0.001, *η*^2^ = 0.33), but no significant main effects of motion direction (*F*(1,18)=0.01, *p*=0.92, *η*^2^ = 0.001) or category (*F*(3,54)=0.1, *p*=0.96, *η*^2^ = 0.005).

Because the three non-identical category conditions (different exemplar, different basic-level category, and different superordinate category) did not differ from each other and showed no effect of motion direction (all *p*s>0.14), they were collapsed into a single “non-identical” condition, analyzed separately for same- and opposite-direction trials (Figure 4). Post-hoc comparisons confirmed that category × motion direction interaction was driven primarily by the identical-exemplar condition. Motion direction significantly affected responses only when the center and surround were identical, with reduced facilitation when the center and surround moved in the same direction compared to opposite directions (*t*(18)=3.49, *p*_FDR_ = 0.003). Moreover, for same-direction trials, SI values were significantly lower for identical exemplars than for the collapsed non-identical conditions (*t*(18)=3.31, *p*_FDR_ = 0.004). This pattern indicates that V1 responses depend jointly on motion direction and stimulus similarity, consistent with sensitivity to contextual structure.

Because center-only responses did not differ significantly from zero in OPA (*t*(18) = −0.90, *p* = 0.38) or PPA (*t*(18) = 0.73, *p* = 0.47), contextual effects in these regions were assessed using PSC comparisons among the center+surround conditions. In both regions, responses were comparable across conditions, and a two-way ANOVA revealed no significant main effects of motion direction (OPA: *F*(1,18)=1.47, *p*=0.24; PPA: *F*(1,18)=0.53, *p*=0.48), category (OPA: *F*(3,54)=0.13 *p*=0.94; PPA: *F*(3,54)=1.5, *p*=0.23), or their interaction (OPA: *F*(3,54)=1.28, *p*=0.29; PPA: *F*(3,54)=1.34, *p*=0.27).

These results indicate that responses in scene-selective regions were not reliably modulated by either motion congruence or categorical similarity at the univariate level under the present stimulus conditions.

### Multivariate analyses reveal motion-dependent contextual modulation in hMT+

To assess how motion direction influences surround modulation, data were collapsed across the four category conditions prior to decoding to isolate motion-related effects. We then decoded the center-only versus center+surround conditions in V1, hMT+, OPA, and PPA under two motion-direction conditions: same-direction and opposite-direction.

**Figure 5:**
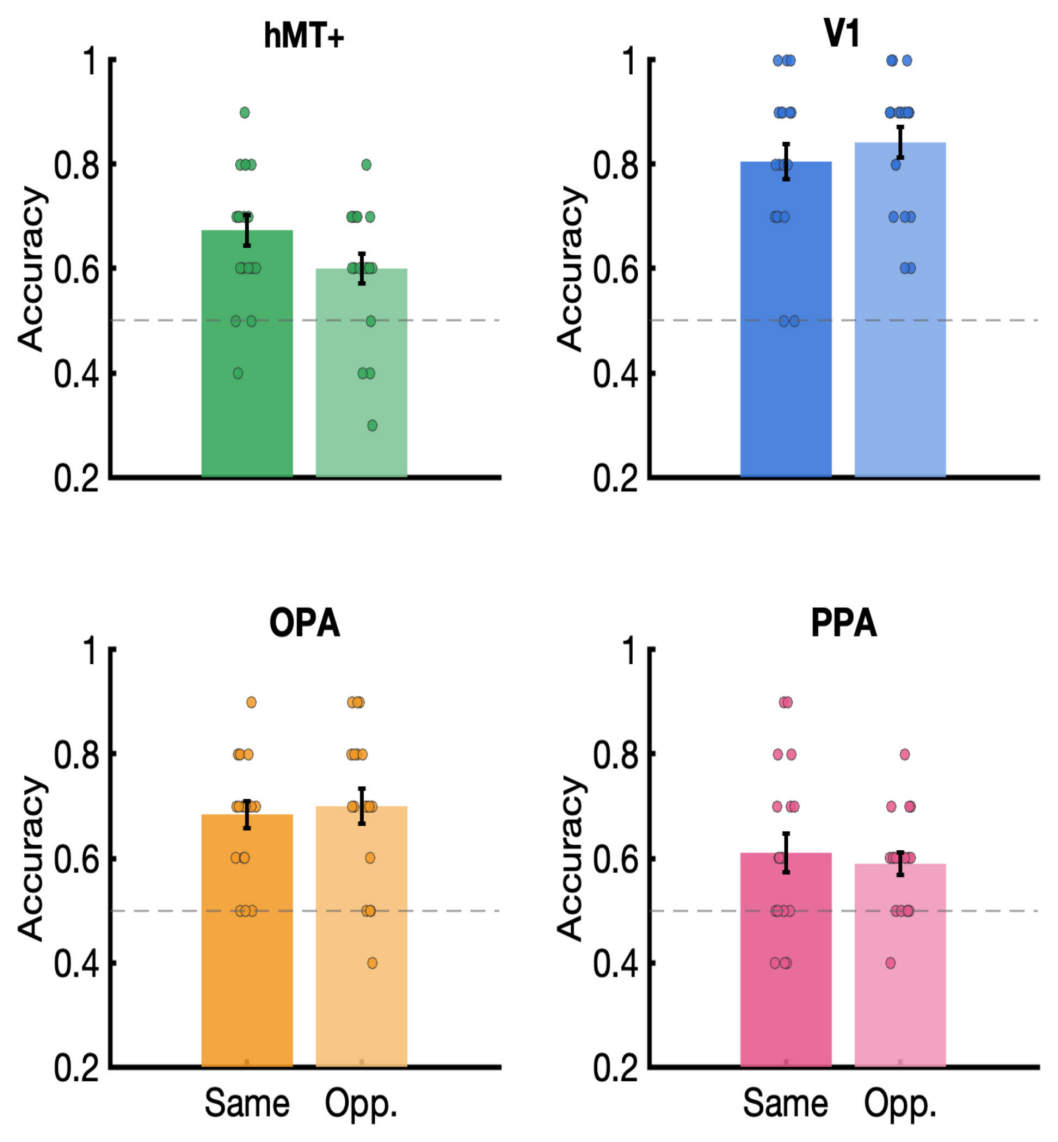
Multivoxel decoding accuracy (N=19) for center-only and center+surround classification for the two motion-direction conditions: same-direction and opposite-direction. Mean decoding accuracy is shown for hMT+, V1, OPA, and PPA. Circles represent individual participant values. Error bars indicate the standard error of the mean (SEM). The dashed line indicates chance-level (0.5).

As shown in Figure 5, decoding accuracy in hMT+ was significantly above chance only for the same-direction condition trials (*p* = 0.02). A paired-samples *t* -test revealed significantly higher decoding accuracy for same- than opposite-direction trials (*t*(18) = 3.07, *p* = 0.007), indicating that motion congruence enhances contextual modulation in hMT+ multivoxel responses. This multivariate pattern closely mirrors the univariate results, which showed stronger contextual suppression when the center and surround moved in the same direction. Together, these analyses demonstrate that hMT+ activity reflects motion-dependent contextual modulation both in overall response amplitude and in distributed activation patterns, consistent with classical motion-dependent modulation observed with simple stimuli [25, 30, 43].

Decoding accuracy was above chance in V1 and OPA for both same-direction and opposite-direction trials (all *p <* 0.05). However, paired-samples *t* -tests revealed no significant differences in decoding accuracy between same- and opposite-direction trials (all *p >* 0.20). As the univariate analysis in V1 revealed a motion-congruence effect only in the identical-exemplar condition, collapsing decoding analyses across category conditions may have reduced sensitivity to detect motion-dependent differences in multivoxel response patterns. Although OPA showed reliable above-chance decoding, the absence of direction-related differences in these regions suggests that their surround modulation reflects general contextual integration rather than motion congruence.

### Multivariate analyses reveal category-dependent contextual representations in OPA and PPA

To assess how category similarity influences surround modulation, data were collapsed across the two motion-direction conditions prior to decoding. We then decoded the center-only versus center+surround conditions in V1, hMT+, OPA, and PPA across four category conditions: identical exemplar, different exemplar, different basic-level category, and different superordinate category.

**Figure 6:**
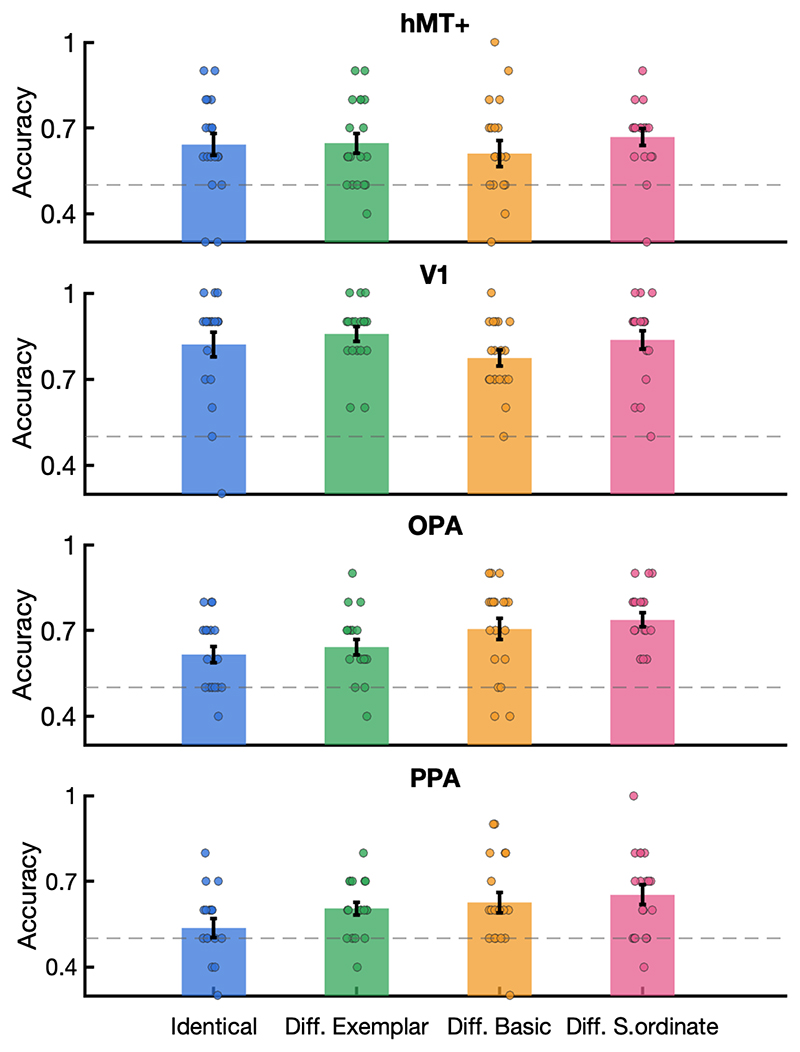
Multivoxel decoding accuracy (N=19) for the center-only versus center+surround classification across the four category conditions: identical exemplar, different exemplar, different basic-level category, and different superordinate category. Mean decoding accuracy is shown for hMT+, V1, OPA, and PPA. Circles represent individual participant values. Error bars represent the standard error of the mean (SEM). The dashed line indicates chance-level (0.5).

As shown in Figures 6, classification accuracy was significantly above chance for all category conditions in V1 (all *p <* 0.05). In hMT+, accuracy was also above chance for all category conditions (all *p <* 0.05) except the different basic-level category condition (*p* = 0.19). Despite robust decoding performance, one-way repeated-measures ANOVAs revealed no effect of category in either region (V1: *F*(3, 54) = 1.93, *p* = 0.13, *η*^2^ = 0.10; hMT+: *F*(3, 54) = 0.56, *p* = 0.64, *η*^2^ = 0.03). These results indicate strong surround modulation in both areas, but this modulation did not depend on categorical similarity between center and surround, consistent with the roles of V1 and hMT+ in processing low-level visual features such as edges, orientation, contrast, and motion rather than higher-level, category-selective information.

As shown in Figure 6, the classification accuracy was significantly above chance in OPA for all category conditions (all *p <* 0.05), except the identical-exemplar condition (*p* = 0.11). A one-way repeated-measures ANOVA revealed a main effect of category, *F*(3, 54) = 4.12, *p* = 0.01, *η*^2^ = 0.19. Post-hoc comparisons showed higher decoding for the different superordinate-category condition than for both the identical- and different-exemplar conditions (all *t*(18) > 3.01, *p <* 0.02), and marginally higher decoding for the different basic-level category condition than for the identical-exemplar condition (*t*(18) = 2.04, *p* = 0.06). Decoding did not differ between the identical- and different-exemplar conditions, *t*(18) = 0.70, *p* = 0.51.

These results suggest that surround modulation in OPA increases with categorical distance. Multivoxel responses were more strongly influenced by the surround when the center and surround scenes belonged to different categories, indicating that OPA is sensitive not only to the presence of contextual surround input but also to its semantic relationship with the center. This categorical sensitivity aligns with our behavioral findings [40], in which surround modulation increased as categorical similarity decreased.

PPA showed a similar pattern, with decoding accuracy increasing as the categorical dissimilarity between center and surround increased. Decoding was above chance for the different basic-level and superordinate category conditions (both *p <* 0.05), marginal for the different exemplar condition (*p* = 0.08), and not significant for the identical-exemplar condition (*p* = 0.33). The main effect of category was significant, *F*(3, 54) = 2.93, *p* = 0.04, *η*^2^ = 0.14. Post-hoc comparisons revealed higher decoding accuracy for the different superordinate-category condition than for the identical-exemplar condition (*t*(18) = 2.59, *p* = 0.02), and higher decoding accuracy for the different basic-level category condition than for the identical-exemplar condition (*t*(18) = 2.18, *p* = 0.04). Compared with OPA, surround modulation in PPA exhibited a more gradual and weaker increase in decoding accuracy with categorical distance. Taken together, these results extend surround modulation effects to the scene-selective cortex, demonstrating that surround modulation in these regions is shaped by higher-level categorical relationships.

To assess whether scene-selective regions reflect contextual relationships between center and surround scenes independent of differences in retinotopic extent, we performed a four-way classification analysis restricted to center+surround trials. This ensured that peripheral stimulation was held constant across conditions. We decoded the category relationship between center and surround scenes (identical exemplar, different exemplar, different basic-level category, and different superordinate category), collapsing across motion-direction conditions to isolate category-related effects.

**Figure 7:**
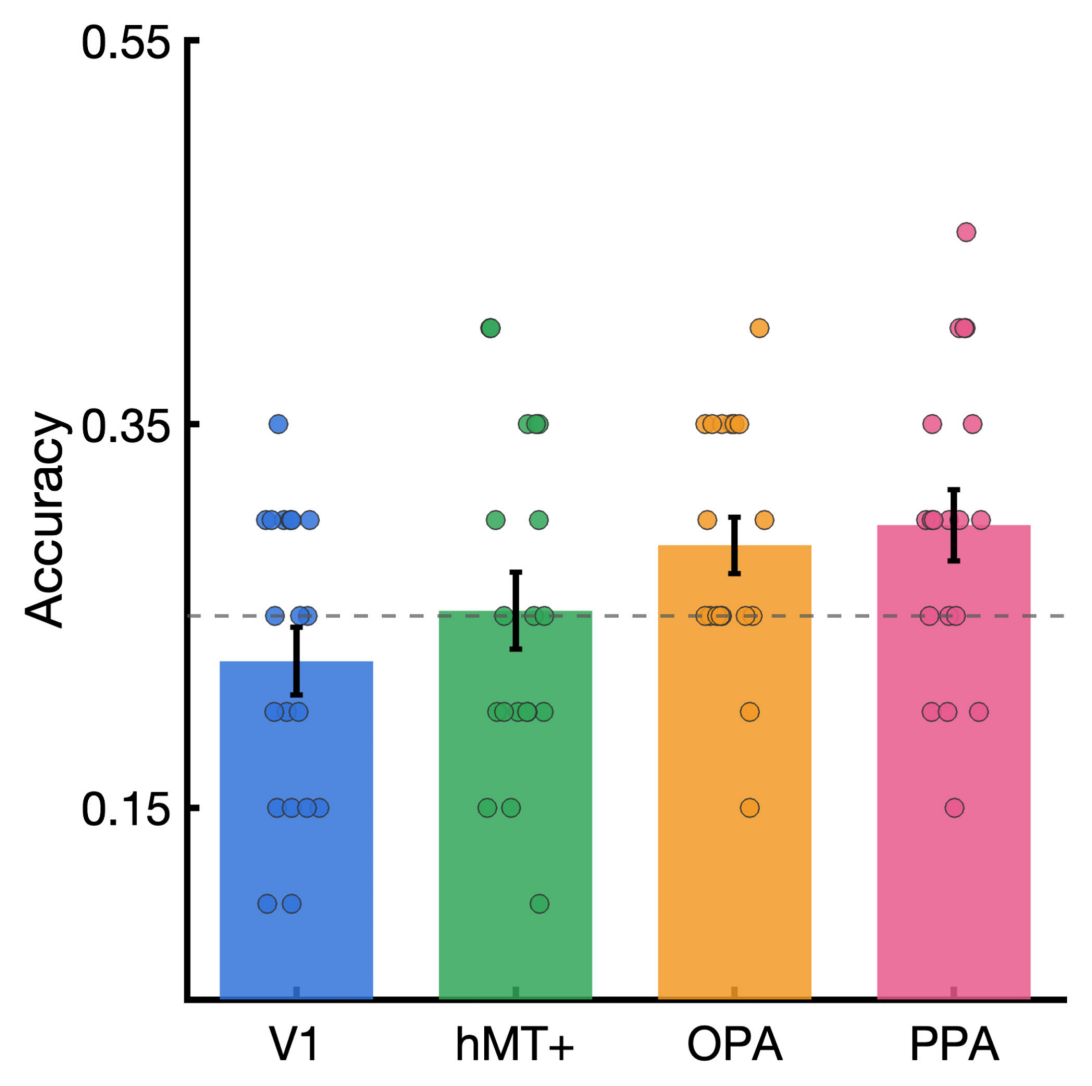
Multivoxel decoding accuracy (N=19) for four-way classification of center–surround category relationships (identical exemplar, different exemplar, different basic-level category, and different superordinate category). Circles represent individual participant values. Error bars represent the standard error of the mean (SEM). The dashed line indicates chance-level (0.25).

As shown in Figure 7, decoding accuracy in V1 and hMT+ did not differ significantly from chance (both *p >* 0.47), indicating that these regions did not reliably represent categorical relationships between center and surround scenes. In contrast, both scene-selective regions showed sensitivity to categorical relationships between center and surround scenes. Decoding accuracy was significantly above chance in both OPA and PPA (both *p <* 0.05). Together, these results suggest that activity patterns in scene-selective cortex reflect contextual relationships between central and surrounding scenes, demonstrating that contextual modulation in these regions is shaped by higher-level categorical structure.

### Visual Feature Analysis

To assess whether the observed effects could be explained by low-level image properties, we conducted a visual feature analysis quantifying low-level visual similarity between center and surround stimuli. Following prior work using hierarchical models of early visual processing [64], we used an HMAX-based representation to characterize low-level visual structure.

Mean low-level similarity between center and surround stimuli was comparable across category conditions (identical exemplar: M = 1; different exemplar: M = 0.93; different basic-level: M = 0.91; different superordinate: M = 0.90). A permutation test revealed no significant effect of categorical condition on similarity (*p* = 0.12). These results indicate that the categorical differences observed in the neural data are unlikely to be explained by systematic differences in low-level visual similarity between center and surround stimuli.

## Discussion

The present study investigated how motion congruence and categorical similarity between center and surround scenes shape neural responses to dynamic natural scenes. Using fMRI, we measured neural responses while systematically varying the contextual relationship between center and surround across four levels of categorical similarity (identical exemplar, different exemplar, different basic-level category, and different superordinate category) and two levels of motion congruence (same vs. opposite direction). Univariate analyses revealed distinct patterns of contextual modulation across visual regions. In hMT+, responses showed robust motion-dependent suppression, with stronger suppression when the center and surround moved in the same direction. In V1, responses varied with the low-level similarity between center and surround stimuli, showing reduced responses (e.g., weaker facilitation) when the two were identical and moved in the same direction. In contrast, scene-selective regions (OPA and PPA) did not show univariate modulation by either motion congruence or categorical similarity. Multivariate analyses complemented these findings. In hMT+, decoding between center-only and center+surround conditions was stronger for same-than opposite-direction motion, indicating enhanced sensitivity to motion congruence at the level of distributed activity patterns. In scene-selective regions, multivoxel patterns distinguished among category conditions, with decoding accuracy increasing as categorical similarity between center and surround decreased, indicating sensitivity to categorical relationships.

These results extend previous work on contextual influences to dynamic natural scenes. While contextual modulation has been extensively characterized using simple stimuli such as gratings [5, 12, 15], its neural basis under naturalistic conditions has remained largely unexplored. The present findings demonstrate that neural responses to natural scenes are shaped by contextual relationships between central and surrounding visual input. Importantly, these contextual effects varied across visual regions, suggesting that contextual influences operate differently depending on the type of information represented in each region. In V1 and hMT+, contextual effects were primarily driven by low-level feature relationships, such as stimulus similarity and motion congruence. In contrast, scene-selective regions showed sensitivity to higher-level contextual structure, as reflected in the categorical relationship between center and surround scenes.

### Motion-dependent contextual modulation in hMT+

hMT+ exhibited stronger suppression when the center and surround moved in the same direction than when they moved in opposite directions. Decoding analyses further confirmed that this contextual modulation was driven specifically by the motion direction, independent of categorical similarity. This pattern aligns with classical neu-rophysiological findings showing that MT neurons show stronger suppression for same-direction motion and weaker or no suppression when the center and surround move in opposite directions [5, 23, 28, 29, 65]. Such motion-dependent modulation likely reflects local inhibitory interactions among direction-selective neurons in MT, consistent with the *MT hypothesis* of surround modulation [12]. By extending these low-level findings to naturalistic conditions, our results provide neural evidence that motion-based center–surround interactions are recruited during complex scene processing in hMT+. Furthermore, the close correspondence between our neural and behavioral findings using identical stimuli [40] suggests that perceptual suppression during natural vision may reflect these motion-sensitive inhibitory mechanisms in hMT+.

### Contextual interaction in V1 reflects physical similarity

In V1, responses varied systematically with the relationship between center and surround stimuli. Specifically, responses were reduced for identical exemplars relative to other category conditions in same-direction trials, indicating that greater low-level similarity between center and surround is associated with weaker responses. Moreover, motion direction affected responses only in the identical-exemplar condition, with lower responses when the center and surround moved in the same direction compared to opposite directions.

This pattern is consistent with classical findings showing stronger suppression for highly similar or homogeneous stimuli, such as iso-oriented gratings [1, 2, 4, 26, 27, 66]. The reduced responses for identical center–surround pairs therefore suggest that V1 is sensitive to low-level similarity between center and surround scenes. The motion-dependent effects also resemble modulation patterns reported in motion-selective cortex. These effects may reflect horizontal interactions within V1 or feedback signals from motion-selective areas such as hMT+, consistent with evidence that motion information can influence early visual processing [19, 67, 68].

Findings from animal studies using naturalistic stimuli provide converging evidence for this interpretation. For example, Coen-Cagli, Kohn, and Schwartz [44] reported that macaque V1 exhibits stronger suppression when the center and surround consist of homogeneous natural image regions. This aligns broadly with our findings, which also reveal contextual modulation driven by physical similarity, although it manifested as reduced facilitation rather than increased suppression. Consistent with this, multivariate analyses further showed robust surround modulation in V1, but this effect did not depend on categorical similarity or motion congruence. Given that the univariate motion effect was observed only in the identical-exemplar condition, collapsing across category conditions in the decoding analysis may have reduced sensitivity to detect motion-dependent differences in multivoxel response patterns.

On the other hand, Onat, Jancke, and König [45] used voltage-sensitive dye imaging in the cat visual cortex to show that V1 responses to natural scene clips were facilitated when the surrounding context was drawn from the same scene. Similarly, a human fMRI study has shown that V1 responses are modulated by the consistency of surrounding scene context, with enhanced responses for scene-coherent compared to incoherent configurations [46]. While this effect is not directly equivalent to our manipulation, it likewise indicates that V1 responses are sensitive to relationships between center and surround in naturalistic stimuli. However, in our study, responses were reduced in the identical (i.e., coherent) condition compared to non-identical conditions. This difference may reflect differences in the contextual manipulations, stimulus properties, and task demands across studies.

The nature of contextual effects in V1 has been shown to vary across studies, with both suppressive and facilitatory influences reported depending on stimulus configuration, task demands, and attentional state [7, 9, 66, 69, 70]. In the present study, responses to the center-only condition were not reliably different from zero, limiting the interpretation of the observed facilitation effects relative to the baseline. One possible explanation for the absence of a reliable center-only response is the nature of the stimuli and task. The central stimulus consisted of natural scene images rather than high-contrast, simple stimuli such as gratings, and was relatively small and briefly presented, which may have produced weaker responses in V1 and a reduced signal-to-noise ratio.

Importantly, the absence of a reliable center-only response does not imply a lack of contextual effects. Univariate responses varied systematically with center–surround similarity, and multivariate analyses further revealed robust surround modulation in V1. Together, these findings indicate that V1 responses were sensitive to center–surround relationships even when the center stimulus alone did not elicit a strong univariate response. Overall, the pattern of results suggests that contextual effects in V1 primarily reflect sensitivity to low-level physical similarity rather than higher-level categorical relationships

### Category-dependent contextual effects in scene-selective cortex

While univariate analyses revealed no category or motion effects, the multivariate analyses showed clear category-dependent contextual effects in scene-selective regions. Both OPA and PPA were sensitive to categorical relationships between center and surround scenes, with multivoxel responses more strongly influenced by the surround when the center and surround belonged to different categories. This pattern was most pronounced in OPA. PPA showed a similar but weaker graded effect with increasing categorical distance, possibly reflecting the lower signal-to-noise ratio typically observed in ventral visual regions [e.g., 71, 72]. Importantly, decoding revealed no motion-related effects in either region, suggesting that response differences in scene-selective cortex are primarily associated with categorical rather than motion-related factors.

The visual feature analysis showed that similarity between center and surround did not differ reliably across non-identical categorical conditions, making it unlikely that these effects are driven by low-level visual features. One potential concern is that the observed effects could instead reflect the recruitment of voxels with retinotopic selectivity for peripheral stimulation. To address this, we conducted an additional analysis restricted to the center+surround conditions, thereby holding the spatial extent of stimulation constant across conditions. This analysis revealed significant decoding across category conditions in both OPA and PPA, indicating that distributed response patterns encode information about center–surround relationships beyond differences in peripheral stimulation. In addition, each surround image appeared equally often across category conditions, making it unlikely that decoding differences reflect stimulus-specific preferences. Together, these findings suggest that the observed effects reflect sensitivity to contextual relationships between central and surrounding scene information rather than differences in peripheral stimulation or low-level visual differences.

Our categorical similarity manipulation aligns with established frameworks of visual categorization that distinguish between superordinate and basic-level representations. Superordinate categories (e.g., indoor vs. outdoor) capture broad semantic distinctions, whereas basic-level categories correspond to more specific and perceptually meaningful scene classes [73, 74]. Converging evidence indicates that such categorical structure is reflected in visual cortex, with coarse distinctions associated with large-scale response differences and finer distinctions relying on more subtle representational variations [74]. Importantly, the three category levels used here establish a clear ordinal structure, such that categorical distance increases monotonically from different exemplars within a basic-level category, to different basic-level categories, and finally to superordinate categories. This graded organization provides a principled and convenient way to parametrize categorical distance [75]. Consistent with this framework, our recent behavioral results, obtained with the same stimulus set and category manipulation [40], revealed a graded increase in contrast thresholds from identical exemplars to different exemplars within the same basic-level category, and further across basic- and superordinate-level categories. This graded pattern is consistent with differences in categorical distance and provides converging support for the validity of our category manipulation. Importantly, these behavioral results are consistent with the present neural findings, indicating that contextual influences vary systematically with categorical dissimilarity.

Similar sensitivity to categorical context has been demonstrated in prior neuroimaging work on scene and object perception. Contextual incongruence between scene elements has been shown to alter neural responses in parahippocampal and occipitotemporal regions [42, 76, 77]. For example, Peyrin et al. [42] reported that categorically congruent (e.g., both man-made) but physically dissimilar (e.g., buildings vs. streets) peripheral scenes disrupted central scene categorization and increased activation in inferior frontal and occipitotemporal cortices. Consistent with this, we observed stronger context-dependent differences even when the center and surround belonged to the same superordinate category (e.g., two indoor scenes) but differed at the basic level (e.g., restaurant vs. museum).

### Contextual influences across visual cortex may support efficient scene representation

Real-world scenes show systematic spatial and semantic regularities (e.g., a bathroom typically contains a sink, mirror, and shower arranged within a characteristic layout). The visual system exploits these regularities to integrate information efficiently across multiple representational levels, supporting rapid understanding of complex environments [34, 36, 78]. Computational and neurophysiological models suggest that such contextual integration relies on recurrent and inhibitory interactions that can modulate sensory input according to its relevance [68, 79, 80]. Consistent with this view, behavioral and neuroimaging work demonstrates that coherent scene structure facilitates both scene and object perception [36, 81–86]. Together, these findings suggest that contextual mechanisms may enhance informative signals while attenuating redundant input, thereby promoting stable and efficient visual processing in natural environments.

Our results extend this framework by showing that categorically incongruent surrounds are associated with stronger contextual modulation in scene-selective cortex than congruent surrounds. Whereas studies using simple stimuli have attributed contextual effects to the suppression of statistical redundancies in low-level features [19, 20, 87–89], the present findings suggest that, at higher representational levels, responses vary systematically with categorical relationships between center and surround. In natural scenes, categorically incongruent information may enhance neural responses because it violates contextual expectations and conveys higher informational value, whereas with simple stimuli, identical information may be suppressed because it is predictable and redundant. In contrast, V1 showed a pattern more consistent with low-level surround modulation, where facilitation decreased when the center and surround were physically identical, indicating a greater influence of visual redundancy than categorical structure. Both forms of modulation may therefore reflect a shared computational principle: optimizing neural representations by emphasizing informative differences while attenuating predictable input. Both forms of modulation may therefore reflect a shared computational principle: optimizing neural representations by emphasizing informative differences while attenuating predictable input.

Together, these results are consistent with a functional differentiation of contextual influences across visual cortex, likely shaped by the functional specialization of distinct visual pathways. Early visual areas (V1) are primarily governed by physical similarity, mid-level motion areas (hMT+) are sensitive to motion congruence, and higher-level scene-selective regions (OPA and PPA) integrate categorical structure. This pattern is broadly consistent with predictive and efficient coding accounts [19, 79, 80], whereby different regions of the visual system may selectively suppress predictable or redundant input and enhance informative discrepancies.

### Limitations and future directions

Although the present study provides neural evidence for category- and motion-dependent contextual effects in natural vision, several questions remain open for future investigation. First, although the visual feature analysis indicated that low-level visual similarity did not differ reliably across categorical conditions, we did not explicitly manipulate low-level image properties such as spatial frequency or amplitude spectrum. It therefore remains possible that more specific or localized visual features, not captured by the present analysis, contribute to surround modulation [42]. Future work could examine these factors more directly by varying physical properties and categorical coherence independently to better disentangle their respective influences.

Second, while we used sub-ROI localizers in V1 and hMT+ to identify voxels corresponding to the center stimulus, this approach could not be applied to OPA and PPA due to their larger receptive fields. Consequently, the ROIs in these regions may have included voxels responsive to both center and surround, so that the effects cannot be directly interpreted as modulatory effects on the representation of the central stimulus. Moreover, scene-selective areas are thought to contain fine-grained functional subfields with heterogeneous tuning to spatial scale, visual field position, and category information [e.g., 38, 90]. The absence of univariate category effects in OPA and PPA may therefore reflect this spatial and functional heterogeneity, where the averaging across voxels with different category or positional preferences could mask category-specific responses. Category-specific modulatory effects may instead depend on multivariate response patterns in scene-selective cortex, necessitating pattern-based analysis approaches for characterizing this fine-scale organization. Category-specific modulatory effects may instead depend on multivariate response patterns in scene-selective cortex, necessitating pattern-based analysis approaches for characterizing this fine-scale organization. As decoding performance alone does not specify which stimulus or response dimensions drive classification, the present results should be interpreted as consistent with categorical similarity effects rather than as definitive evidence for a specific representational mechanism.

Moreover, although our stimuli consisted of natural scenes, the applied drifting motion does not fully reflect the complexity of motion in everyday visual experience. While coherent motion can occur during self-motion or eye movements, natural environments typically involve more complex and heterogeneous motion patterns. Thus, our stimuli provide a controlled approximation of naturalistic motion, and future work should extend this approach using more complex, dynamic videos to better capture these dynamics.

Finally, the relatively small central aperture (1.9°) may have limited the amount of scene information available and potentially constrained explicit recognition of detailed scene content. Although similar stimulus sizes are commonly used in studies of center–surround interactions [12, 15], and prior work demonstrates that meaningful scene information can be extracted from comparable visual extents [41, 91], the present study did not include a direct measure of perceptual scene categorization. However, the stimuli and design closely matched those used in our recent behavioral study [40], in which participants reliably categorized central scenes and exhibited robust contextual effects under comparable conditions, providing indirect evidence that the central stimuli conveyed perceptually relevant information. Future work combining neuroimaging with concurrent behavioral measures will be important to more directly establish the relationship between neural responses and perceptual experience.

## Conclusions

Using dynamic natural scenes, we show that contextual influences on neural responses depend on both motion-direction congruence and categorical relationships between center and surround, but that these effects differ systematically across functionally distinct visual regions. In hMT+, univariate and multivariate analyses revealed motion-dependent contextual modulation, with stronger effects for same-direction than opposite-direction motion, consistent with classical center–surround interactions in motion-selective cortex. In V1, responses were sensitive to low-level similarity between center and surround, with reduced responses when the two were physically identical and moved in the same direction. In scene-selective cortex (OPA and PPA), multivoxel activity patterns encoded the categorical relationship between center and surround scenes, with decoding accuracy increasing as categorical similarity between center and surround decreased. Together, these findings indicate that contextual modulation on natural scene processing differs across cortical regions, shifting from sensitivity to low-level feature relationships in early and motion-selective cortex to sensitivity to higher-level categorical structure in scene-selective regions. This reflects the distinct representational roles of these regions and suggests that contextual influences operate at multiple levels of visual representation. More broadly, this pattern is consistent with predictive and efficient coding accounts, whereby different regions of the visual system attenuate predictable input and emphasize informative discrepancies to support perceptual stability and efficient scene understanding.

## Data Availability

The data that support the findings of this study are available at the following DOI: (https://doi.org/10.5281/zenodo.17774893).

## New & Noteworthy

Visual perception depends on contextual interactions across the visual field. Using fMRI and dynamic natural scenes, we show that contextual modulation differs across the visual system: it is motion-dependent in hMT+, reflects low-level similarity in V1, and is shaped by categorical relationships between center and surround in scene-selective cortex. Together, these findings reveal differences across functionally distinct visual regions in how contextual information is represented during natural vision.
